# Circulating metabolites and depression: a bidirectional Mendelian randomization

**DOI:** 10.3389/fnins.2023.1146613

**Published:** 2023-04-21

**Authors:** Yankai Dong, Zengxiao Zou, Pin Deng, Xiaoping Fan, Chunlin Li

**Affiliations:** ^1^Department of Cardiovascular Surgery, Guangdong Provincial Hospital of Chinese Medicine, The Second Affiliated Hospital of Guangzhou University of Chinese Medicine, Guangzhou, Guangdong, China; ^2^The Second Clinical College of Guangzhou University of Chinese Medicine, Guangzhou, Guangdong, China; ^3^Department of Hand and Foot Surgery, Beijing University of Chinese Medicine Third Affiliated Hospital, Beijing, China; ^4^Department of Neurology, Affiliated Hospital of Shandong University of Traditional Chinese Medicine, Jinan, Shandong, China

**Keywords:** circulating metabolites, depression, Mendelian randomization, instrumental variable, bidirectional

## Abstract

**Background:**

Studies have shown an association between depression and circulating metabolites, but the causal relationship between them has not been elucidated. The purpose of this study was to elucidate the causal relationship between circulating metabolites and depression and to explore the role of circulating metabolites in depression.

**Methods:**

In this study, the top single-nucleotide polymorphisms (SNPs) associated with circulating metabolites (*n* = 24,925) and depression (*n* = 322,580) were obtained based on the publicly available genome-wide association study using two-sample Mendelian randomization (MR). SNP estimates were summarized through inverse variance weighted, MR Egger, weighted median, MR pleiotropy residual sum and outlier, and “leave-one-out” methods.

**Results:**

Apolipoprotein A-I (OR 0.990, 95% CI 981–0.999) and glutamine (OR 0.985, 95% CI 0.972–0.997) had protective causal effects on depression, whereas acetoacetate (OR 1.021, 95% CI 1.009–1.034), glycoproteins (OR 1.005, 95% CI 1.000–1.009), isoleucine (OR 1.013, 95% CI 1.002–1.024), and urea (OR 1.020, 95% CI 1.000–1.039) had an anti-protective effect on depression. Reversed MR showed no effect of depression on the seven circulating metabolites.

**Conclusion:**

In this study, MR analysis showed that apolipoprotein A-I and glutamine had a protective effect on depression, and acetoacetate, glycoprotein, isoleucine, glucose, and urea may be risk factors for depression. Therefore, further research must be conducted to translate the findings into practice.

## 1. Introduction

Depression is a common mental disorder. The main clinical feature of depression is obvious and lasting despondent. Depression is associated with high rates of morbidity, disability, and mortality, which brings serious harm to the physical and mental health of patients (Krishnan and Nestler, 2008; Yuan et al., 2020; Chen L. et al., 2022), and ~300 million people worldwide suffer from the disease (Herrman et al., 2019). The economic burden of depression is ~ $2.5 trillion, which is 10% of the total global burden of disease (Tran et al., 2020). Depression has complex pathogenesis, involving the hypothalamic–renal gland, the hypothalamic–pituitary–adrenal axis, genetics, body metabolism, neurotrophic factors, and other influencing factors, and it is affected by the physiological, biochemical, social environment, and many other aspects (Liu et al., 2015).

Metabolomics, a branch of systems biology, is a new omics technology developed after proteomics, genomics, and transcriptomics. It has shown great advantages in the field of disease diagnosis (Brindle et al., 2002; German et al., 2004). The application of metabolomics in depression is relatively late. Moreover, given the different instruments and equipment used and the different experimental objects studied, the conclusions obtained by metabolomics in the studies of depression are different, and differences are observed on whether different metabolites play an important role in depression. For example, studies have shown inconsistent expression levels of valine (Liu et al., 2015; Zheng et al., 2017), leucine (Shi et al., 2013; Li D. et al., 2020; Li Y. et al., 2020), glutamine (Du et al., 2017; Geng et al., 2020; Li D. et al., 2020), tryptophan (Zheng et al., 2017; Gui et al., 2018), and tyrosine (Liu et al., 2016; Kawamura et al., 2018) in the blood of patients or animals with depression. Based on the abovementioned research, changes in circulating metabolites may contribute to depression. For the clinical treatment of depression, whether the correlation is causal must be determined, and the most important metabolites must be identified. In addition, some metabolites that play important roles in depression may be undetected because of testing equipment, technology, and the sample itself.

Through a genome-wide association study (GWAS), the causal relationship between exposure phenotype and outcome phenotype can be powerfully and effectively established by using Mendelian randomization (MR) (He et al., 2021; Zhao et al., 2021). These genetic variants are fixed at conception, helping to rule out potential confounding factors and prevent reverse causation (Davies et al., 2018). Compared to observational studies, MR studies can effectively make effective and accurate causal inference (Bowden and Holmes, 2019). In this study, MR was used to explore and clarify the causal effect of circular metabolites on depression and tested whether the effect is bidirectional.

## 2. Materials and methods

**Figure 1:**
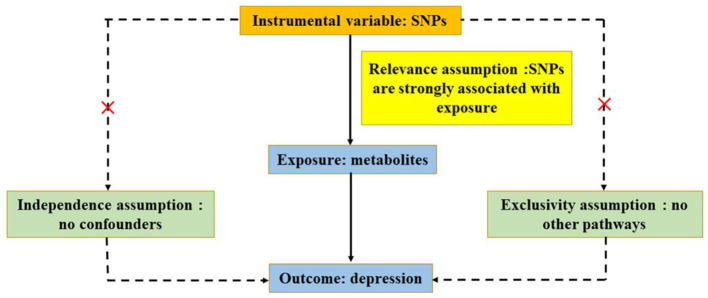
Three significant assumptions of metabolites on depression *via* forward MR. The three paths represent these three different assumptions. Relevance assumption: SNPs are associated with metabolites (the exposure). Independence assumption: SNPs are completely independent of any potential confounding factors that influence metabolites and depression. Exclusivity assumption: SNPs affect metabolites only through metabolites (exposure) and not *via* any alternative causal pathways.

**Figure 2:**
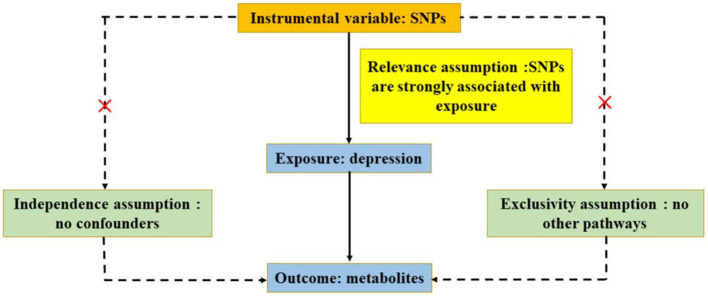
Three significant assumptions of depression on metabolites *via* reverse MR. The three paths represent these three different assumptions. Relevance assumption: SNPs are associated with depression (the exposure). Independence assumption: SNPs are completely independent of any potential confounding factors that influence depression and metabolites. Exclusivity assumption: SNPs affect metabolites only through depression (exposure) and not *via* any alternative causal pathways.

On the basis of publicly available GWAS, summary-level data were acquired (Chen L. et al., 2022). Data on circulating metabolites provided by GWAS were carried out by the University of Oulu (Kettunen et al., 2016), and the data on depression provided by GWAS were primarily performed by the University of Edinburgh (Howard et al., 2018). GWAS released the summary-level data for further analysis, and each cohort involved in the research acquired a consent form of participants and ethical approval. In ensuring effective MR analysis, three important assumptions must be demonstrated: (1) SNPs are associated with circulating metabolites (depression); (2) SNPs affect depression (circulating metabolites) only through circulating metabolites (depression) and not through any other causal pathway, and (3) SNPs are completely independent of any potential confounding factors affecting circulating metabolites and depression (Figures 1, 2).

### 2.1. Circulating metabolites

Instrumental variables (IVs) for circulating metabolites were obtained based on a large-scale study containing 14 cohorts from Europe with a total of 24,925 participants. The sources of the cohorts were as follows: (1) Estonian Genome Center of University of Tartu Cohort, (2) Erasmus Rucphen Family Study, (3) A subsample of FINRISK 1997, (4) Finnish Twin Cohort, (5) Genetics of METabolic Syndrome, (6) Helsinki Birth Cohort Study, (7) Cooperative Health Research in the Region of Augsburg, (8) Leiden Longevity Study, (9) Northern Finland Birth Cohort 1966, (10) Netherlands Twin Register, (11) FINRISK subsample of incident cardiovascular cases and controls, (12) EGCUT sub-cohort, (13) The Cardiovascular Risk in Young Finns Study, and (14) Genetic Predisposition of Coronary Heart Disease in Patients Verified with Coronary Angiogram. The included cohorts were conducted in Estonia, Finland, Netherlands, and Germany. The mean age of participants in the 14 cohorts ranged from 23.9 ± 2.1 to 61.3 ± 2.9, and the mean BMI ranged from 23.1 ± 3.7 to 28.2 ± 4.8. In addition, the proportion of female subjects ranged from 37 to 64%. Using the same platform for each cohort, metabolites that represent a wide range of molecular features of whole-body metabolism were quantified. A variety of metabolic pathways were covered by the metabolite set. Most metabolomic analyses were conducted using an integrated quantitative platform. The genomic location used in the research is human genome construction. Analyzing each cohort separately and in the fixed-effect meta-analysis, a two-genome control correction was used to combine exact interpolation with SNPs with minor allelic counts >3, that is, the individual cohort results and the meta-analysis results were corrected for the genome bloat factor implemented in GWAMA. After screening and meta-analysis, the final results considered variations found in more than seven studies. The genome-wide significance level was set at 2.27^*^10^−9^, correcting for 22 independent tests because the metabolite data were correlated (the standard genome-wide significance threshold was 5^*^10^−8^/22, and the number of major components accounted for more than 95% of the variance in the metabolomics data). The number of independent tests was derived from the number of major components that accounted for more than 95% of the variation in the metabolite data. The genome bloat factor for all traits in the meta-analysis was <1.034, which indicated that there was no systematic bias in test statistics.

### 2.2. Depression phenotypes

Summary statistics were obtained from large publicly available GWAS of depression. For the depression phenotypes, the exclusion would be applied to participants if they were identified with schizophrenia, bipolar disorder, or personality disorder through touchscreen responses, self-declared data, or ICD codes from hospital admission records; participants were excluded if they reported having a prescription for an antipsychotic medication during a verbal interview. Control individuals would be excluded if they had reported having a prescription for antidepressants, had a diagnosis of a depressive mood disorder from hospital admission records, or had self-reported depression. From the NHS National Research Ethics Service, this study was performed under generic approval. Full informed written consent was given to all participants. Based on the conventional threshold of *P* < 5 × 10^−8^, genome-wide statistical significance was determined (Howard et al., 2018).

### 2.3. Statistical analysis

Using the package twosampleMR (Yuan et al., 2022) in R software, all analyses were conducted. According to the *P*-value of 5 × 10^−8^, the SNPs (IVs) in the GWAS were identified. In addition, based on a correlation index R^2^ ≤ 0.001 and 10,000 kilobases apart for distance cutoff, these SNPs were uncorrelated (if there are fewer than two SNPs, then adjust the *P*-value of 5 × 10^−6^ (Wang et al., 2020) and correlation index R^2^ ≤ 0.01 and 5,000 kilobases apart for distance cutoff). On the basis of the GWAS summary statistics, the effect estimates and corresponding standard errors (SE) of these SNPs of circulating metabolites and depression were acquired. The data for exposure (circulating metabolites) and outcome (depression) were harmonized, and palindromic SNPs were removed (Chen L. et al., 2022). Using simex package in R software and the formula *F* = total R^2^ × (N – 1 – K) / [(1 – total R^2^) × K], where K represents the amount of IVs, N represents the sample size, total R^2^ represents the proportion of variance in the exposure explained by the genetic variants, and by calculating the F-statistic, the strength of IVs was assessed. No significantly weak instrumental bias (Li et al., 2022) was observed when the F-statistic was >10. The inverse-variance weighted (IVW) model was applied as the major statistical method (Chen L. et al., 2022). Using sensitivity analyses, such as the weighted median, MR-Egger (Wang et al., 2022), and MR-pleiotropy residual sum and outlier (MR-PRESSO) (Verbanck et al., 2018; Yang et al., 2020), the possible pleiotropy was examined. The weighted median method assumes that more than 50% of the weight comes from SNPs and provides a consistent estimate of causal effects. Using the formula β = ln (OR), the estimated combined effect β was converted into odds ratio (OR). The effect size and corresponding SE for each circulating metabolite (depression) were calculated through MR analysis. The results were presented as

OR and 95% confidence interval (CI). Thereafter, a scatter plot was created to visually examine the potential pleiotropy by showing the causal effects of each SNP of metabolite on depression. With Cochran's Q statistics, the heterogeneity of analysis results was examined, and there was significant heterogeneity in SNP effect estimation when *P* < 0.1 (Chen M. et al., 2022). Using the “leave-one-out” method, sensitivity analysis was conducted in our study. In other words, after establishing the causal relationship, no heterogeneity or pleiotropy appeared during analysis, and each relevant SNP was deleted one by one. In assessing the impact of each SNP, the aggregate effect of the remaining SNPs was calculated (Wang et al., 2022).

## 3. Results

### 3.1. Instrumental variables for circulating metabolites on depression

Based on the selection criteria of IVs, a total of 330 SNPs and 13 SNPs were used as IVs for 35 circulating metabolites and depression, respectively. The SNP characteristics and F-statistic for each circulating metabolite and depression are shown in Supplementary Tables 1, 2. The strength of each circulating metabolite has an F-statistic value between 22.33 and 250.28, and the F-statistic value for depression is 38.63, eliminating the bias of weak IVs.

### 3.2. Causal effect of circulating metabolites on depression

**Figure 3:**
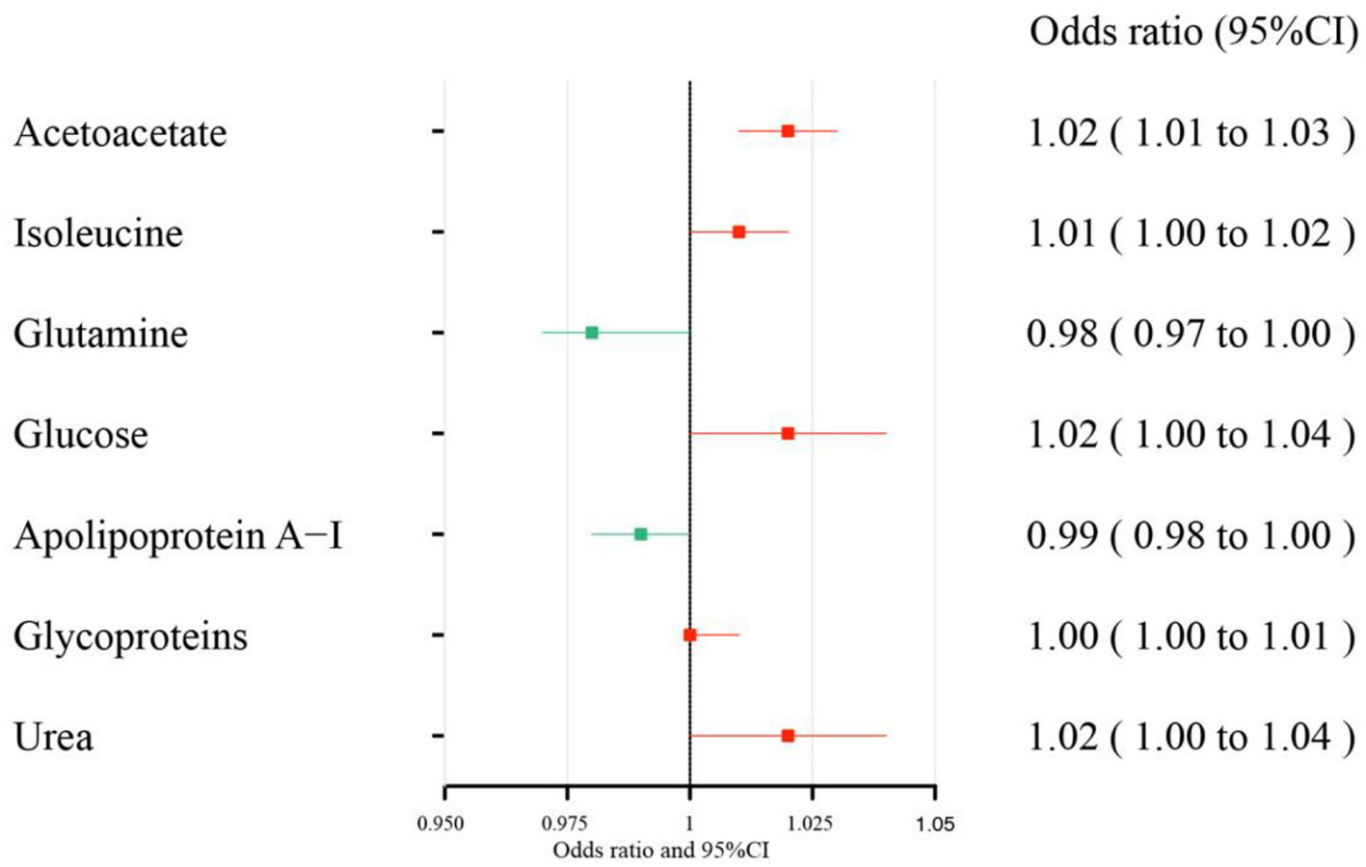
Effect size of each metabolite on depression. There is a protective causal effect when the OR value is <1, whereas a causal pathogenic impact appears when the value is >1. Through the IVW and weighted median analysis, the results were generated.

**Figure 4:**
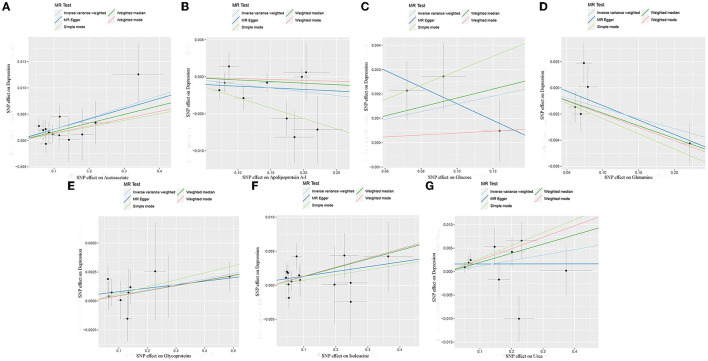
Scatter plots of each metabolite associated with depression. **(A)**, Acetoacetate; **(B)**, Apolipoprotein A-I; **(C)**, Glucose; **(D)**, Glutamine; **(E)**, Glycoproteins; **(F)**, Isoleucine; **(G)**, Urea.

The MR estimates of circulating metabolites on depression are shown in Table 1, Figures 3, 4. Among these 35 metabolites, seven metabolites with significant properties were screened using IVW or weighted median. The result showed that glutamine (OR 0.985, 95% CI 0.972–0.997; *P*-value = 0.019) and apolipoprotein A-I (OR 0.990, 95% CI 0.981–0.999; *P*-value = 0.036) were negatively associated with depression, indicating a protective effect on depression. Acetoacetate (OR 1.022, 95% CI 1.009–1.034; *P*-value = 0.0008), isoleucine (OR 1.013, 95 % CI 1.003–1.024; *P*-value = 0.016), glucose (OR 1.019, 95% CI 1.001–1.036; *P*-value = 0.034), glycoproteins (OR 1.005, 95% CI 1.000–1.009; *P*-value = 0.038), and urea (OR 1.020, 95% CI 1.000–1.039; *P*-value = 0.044) were positively associated with depression. The causal effects of each genetic variation of each circulating metabolite on depression are shown in Supplementary Figure 1.

**Table 1:** Features of the included study population.

**Exposure**	**Methods**	**Number of SNPs**	**OR (95% CI)**	**Beta (SE)**	***P*-value**	**Fdr**	**Pleiotropy**	**Heterogeneity**	
							***P-*** **value**	*I*^2^ **(%)**	***P-*** **value**
22:6, docosahexaenoic acid	IVW	6	0.996 (0.982–1.010)	−0.004(0.007)	0.577	0.232	0.862	38.324	0.150
3-hydroxybutyrate	IVW	9	1.008 (0.989–1.028)	0.008(0.010)	0.400	0.197	0.816	45.775	0.064
Acetate	IVW	13	1.013 (0.995–1.031)	0.013(0.009)	0.159	0.114	0.739	52.699	0.013
Acetoacetate	IVW	12	1.021 (1.009–1.034)	0.021(0.006)	0.001	0.011	0.901	0.000	0.871
Alanine	IVW	6	1.013 (0.998–1.028)	0.013(0.007)	0.079	0.086	0.930	19.326	0.287
Apolipoprotein A-I	IVW	10	0.990 (0.981–0.999)	−0.010(0.005)	0.036	0.069	0.666	34.134	0.135
Apolipoprotein B	IVW	16	0.999 (0.993–1.006)	−0.001(0.003)	0.834	0.283	0.071	15.222	0.279
Citrate	IVW	5	0.997 (0.977–1.017)	−0.003(0.010)	0.767	0.266	0.896	55.540	0.061
Creatinine	IVW	6	1.000 (0.984–1.016)	0.00007(0.008)	0.993	0.319	0.997	14.615	0.321
Free cholesterol	IVW	11	0.995 (0.989–1.001)	−0.005(0.003)	0.095	0.090	0.347	0.000	0.923
Glutamine	IVW	5	0.985 (0.972–0.997)	−0.016(0.007)	0.019	0.061	0.626	26.614	0.244
Glycerol	IVW	15	1.003 (0.994–1.011)	0.003(0.004)	0.537	0.226	0.367	0.000	0.972
Glycoprotein acetyls	IVW	7	1.007 (0.995–1.020)	0.007(0.006)	0.248	0.152	0.706	22.283	0.259
Glycoproteins	IVW	10	1.005 (1.000–1.009)	0.005(0.002)	0.038	0.070	0.511	0.000	0.944
Histidine	IVW	5	0.992 (0.971–1.015)	−0.008(0.011)	0.501	0.219	0.545	56.649	0.056
Isoleucine	IVW	14	1.013 (1.002–1.024)	0.0132(0.005)	0.016	0.058	0.454	0.000	0.697
Lactate	IVW	12	1.011 (0.998–1.023)	0.011(0.006)	0.096	0.090	0.820	0.967	0.434
Leucine	IVW	3	1.005 (0.988–1.021)	0.005(0.008)	0.574	0.232	0.410	0.000	0.411
Linoleic acid	IVW	14	1.002 (0.995–1.008)	0.002(0.003)	0.621	0.239	0.577	17.890	0.258
Omega-3 fatty acids	IVW	6	0.997 (0.983–1.011)	−0.003(0.007)	0.674	0.246	0.675	47.792	0.088
Omega-6 fatty acids	IVW	12	0.995 (0.988–1.002)	−0.005(0.005)	0.167	0.117	0.541	0.000	0.702
Phenylalanine	IVW	3	0.994 (0.960–1.029)	−0.006(0.018)	0.727	0.256	0.946	74.658	0.019
Phosphatidylcholine and other cholines	IVW	6	0.995 (0.987–1.004)	−0.005(0.004)	0.266	0.158	0.339	0.000	0.674
Total lipids in small LDL	IVW	19	0.997 (0.992–1.003)	−0.003(0.003)	0.314	0.174	0.123	0.000	0.526
Pyruvate	IVW	19	1.009 (0.999–1.019)	0.009(0.005)	0.081	0.086	0.337	40.173	0.037
Serum total cholesterol	IVW	20	0.996 (0.990–1.002)	−0.004(0.003)	0.154	0.113	0.520	0.000	0.822
Serum total triglycerides	IVW	12	1.008 (0.998–1.019)	0.008(0.005)	0.107	0.092	0.505	46.574	0.038
Sphingomyelins	IVW	7	0.992 (0.982–1.001)	−0.008(0.005)	0.083	0.087	0.186	0.000	0.663
total fatty acid	IVW	11	1.004 (0.995–1.013)	0.004(0.004)	0.340	0.182	0.716	16.218	0.289
Total phosphoglycerides	IVW	8	0.997 (0.989–1.005)	−0.003(0.004)	0.482	0.215	0.411	0.000	0.846
Valine	IVW	5	1.003 (0.989–1.016)	0.003(0.007)	0.684	0.248	0.323	0.000	0.461
Mono-unsaturated fatty acids	IVW	7	1.004 (0.994–1.015)	0.004(0.006)	0.419	0.202	0.899	25.567	0.234
Tyrosine	IVW	4	1.003 (0.989–1.018)	0.003(0.007)	0.634	0.241	0.751	0.000	0.813
Glucose	Weighted median	3	1.018 (1.001–1.036)	0.018(0.009)	0.033	0.068	0.372	29.955	0.240
Urea	Weighted median	9	1.020 (1.000–1.039)	0.020(0.010)	0.044	0.071	0.318	30.467	0.175

### 3.3. Evaluation of assumptions and sensitivity analyses

Horizontal pleiotropy was not observed in the intercept of MR Egger regression (acetoacetate, *P* = 0.901; apolipoprotein A-I, *P* = 0.666; glucose, *P* = 0.372; glutamine, *P* = 0.626; glycoproteins, *P* = 0.511; isoleucine, *P* = 0.454; urea, *P* = 0.318), which further showed that the causal effect was not biased by pleiotropy. In addition, no heterogeneity was observed in our study (acetoacetate, *P* = 0.871, I^2^ = 0.000%; apolipoprotein A-I, *P* = 0.135, I^2^ = 34.134%; glucose, *P* = 0.240, I^2^ = 29.955%; glutamine, *P* = 0.244, I^2^ = 26.614 %; glycoproteins, *P* = 0.944, I^2^ = 0.000%; isoleucine, *P* = 0.697, I^2^ = 0.000%; urea, *P* = 0.175, I^2^ = 30.467%; Table 1).

**Figure 5:**
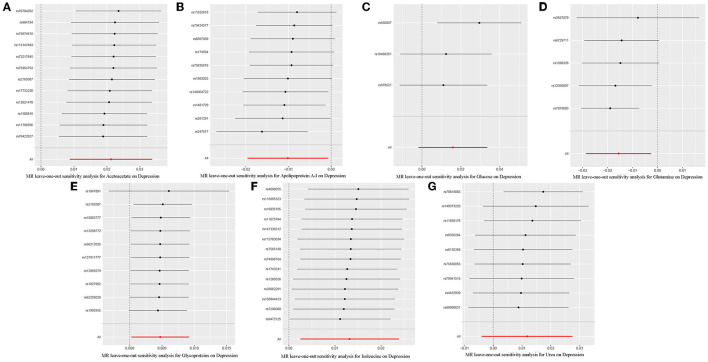
Leave-one-out plot to visualize the causal effect of each metabolite associated with depression when leaving one SNP out. **(A)**, Acetoacetate; **(B)**, Apolipoprotein A-I; **(C)**, Glucose; **(D)**, Glutamine; **(E)**, Glycoproteins; **(F)**, Isoleucine; **(G)**, Urea.

Through leave-one-out analysis, each SNP effect on the overall causal estimate was verified. After removing each SNP, MR analysis was systematically performed again on the remaining SNPs (Figure 5). The results remained consistent, indicating a significant causal relationship among the calculated results of all SNPs. In addition, no dominant SNPs were observed in circulating metabolite levels, and the previous MR results were valid. Moreover, except for glucose and glutamine, funnel plots of the remaining circulating metabolites showed roughly symmetrical causal effects (Supplementary Figure 2).

### 3.4. Causal effect of depression on circulating metabolites

Reverse MR analyses were performed, and the results indicated that depression had no causal effect on acetoacetate (OR = 1.178, 95% CI = 0.534–2.599, *P* = 0.685), apolipoprotein A-I (OR = 0.800, 95% CI = 0.358–1.782, *P* = 0.585), glucose (OR = 1.028, 95% CI = 0.497–2.127, *P* = 0.940), glutamine (OR = 0.569, 95% CI = 0.276–1.170, *P* = 0.125), glycoproteins (OR = 1.982, 95% CI = 0.873–4.501, *P* = 0.102), isoleucine (OR = 1.614, 95% CI = 0.791–3.293, *P* = 0.188), and urea (OR = 1.349, 95% CI = 0.545–3.338, *P* = 0.518). An MR–Egger intercept was performed, and the test showed no horizontal pleiotropy (*P* > 0.05). Furthermore, Cochran's Q statistics showed no heterogeneity (*P* > 0.05), as shown in Table 2.

**Table 2:** Effect of depression on circulating metabolites.

**Exposure**	**Outcome**	**Methods**	**Number of SNPs**	**OR (95% CI)**	**Beta (SE)**	***p-*value**	**Fdr**	**Pleiotropy**	**Heterogeneity**	
								***P-*** **value**	*I*^2^ **(%)**	***P-*** **value**
Depression	Acetoacetate	IVW	13	1.178 (0.534–2.599)	0.164 (0.404)	0.685	0.702	0.581	0.000	0.734
	Apolipoprotein A-I	IVW	13	0.800 (0.358–1.782)	−0.223(0.409)	0.585	0.668	0.759	0.000	0.852
	Glucose	IVW	13	1.028 (0.497–2.127)	0.028 (0.371)	0.940	0.764	0.978	0.000	0.540
	Glutamine	IVW	13	0.569 (0.276–1.170)	−0.564(0.368)	0.125	0.339	0.596	0.000	0.933
	Glycoproteins	IVW	13	1.982 (0.873–4.501)	0.684 (0.419)	0.102	0.339	0.542	0.000	0.780
	Isoleucine	IVW	13	1.614(0.791–3.293)	0.479(0.364)	0.188	0.393	0.397	0.000	0.886
	Urea	IVW	13	1.349(0.545–3.338)	0.299 (0.462)	0.518	0.641	0.164	22.526	0.216

## 4. Discussion

The pathological process of diseases could be revealed by conducting quantitative analysis of all metabolites in organisms, analyzing and comparing small-molecule metabolites *in vivo* under different pathophysiological states, comprehensively monitoring multiple metabolic pathways related to diseases, and then searching for valuable biomarkers. The in-depth study of metabolite-level biomarkers in patients with depression can elucidate the pathogenesis of depression and propose effective treatment plans. However, observational studies are widely used to investigate the relationship between phenotypes and disease, but they cannot be used to study exposure to causation (Kou et al., 2020). Based on extensive GWAS data on circulated metabolites (exposure) and depression (outcomes), this study is the first to conduct a two-sample bidirectional MR analysis as well as explore and elucidate the causal relationship between circulating metabolites and depression. Our research found that glutamine and apolipoprotein A-I were negatively associated with depression, which indicated that glutamine and apolipoprotein A-I had a protective effect against depression. In addition, a possible positive association of acetoacetate, isoleucine, glucose, glycoproteins, and urea with depression was found, which indicated that these five metabolites were risk factors for depression. Using MR-PRESSO and leave-one-out analysis, our results were examined and showed consistent findings.

Compared with previous studies, this study found that docosahexaenoic acid (22:6) (Hoge et al., 2019), omega-3 fatty acids (Hoge et al., 2019; De Sousa and dos Santos, 2022), omega-6 fatty acids (De Sousa and dos Santos, 2022), tyrosine (Islam et al., 2020), phenylalanine (Islam et al., 2020), valine (Baranyi et al., 2016), leucine (Whipp et al., 2022), linoleic acid (Li D. et al., 2020), 3-hydroxybutyrate (Saito et al., 2021) and acetate (Huang et al., 2021) were not associated with depression probably because the research level is different. Previous research conducted detection based on physiological indicators of the body, but this study uses SNPs as an IV, thereby reducing the interference of potential influencing factors, such as instruments, equipment, operation, sampling, and other factors.

An unbiased detection of the causal effects of circulating metabolites on depression was provided on the basis of our MR study. Using genetic variants as IVs, seven metabolites associated with depression risk were observed, including acetoacetate (*P* = 0.000853), isoleucine (*P* = 0.015693), glutamine (*P* = 0.018849), glucose (*P* = 0.033466), apolipoprotein A-I (*P* = 0.035992), glycoproteins (*P* = 0.037874), and urea (*P* = 0.043872). As we have seen, this is the first MR research that combined metabolomics with genomics to evaluate the causal effects of circulating metabolites on depression. Novel insight to uncover the functions of genetic-environmental interactions in the occurrence and development of human diseases was proposed in our study.

At present, the effect of acetoacetate on depression still needs further research. The research of Hou, LJ et al. suggested that acetoacetate was identified as a potential biomarker for diagnosing depression in HBV-infected patients (Hou et al., 2015). In addition, acetoacetate was associated with depressive behavior induced by CUMS (Wu et al., 2015). The results indicated that adjusting the level of acetoacetate in patients with depression might be an effective method for treating depression. Research showed that acetoacetate in the hippocampus could be significantly increased by the intraventricular application of acetoacetate, and then the hippocampus neural inflammation was inhibited, and neurotrophic factors were promoted. Apart from acting as an energy substrate, acetoacetate can also protect neurons by promoting BDNF expression and inhibiting hippocampal nerve inflammation (Wu et al., 2022). Miyamoto et al. found that acetoacetate is also a ligand for GPR43 (Miyamoto et al., 2019), and acetoacetate-GPR43 coupling can inhibit pERK and its two substrates, namely, IL-6 and TNF-α (Wu et al., 2022). Massieu et al. demonstrated that in *in vivo* and *in vitro* experiments, acetoacetate protected hippocampal neurons from the neurotoxicity of glutamate after the application of glycolysis inhibitors (Massieu et al., 2003).

Isoleucine plays various physiological functions as a branched amino acid (Zhang et al., 2017). Koochakpoor et al. observed an inverse relationship between isoleucine intake and risk for depression (Koochakpoor et al., 2021). In addition, a decrease in the sum of isoleucine was observed in depression patients induced by interferon-α (Baranyi et al., 2013). Furthermore, another study found lower isoleucine levels in depression (Baranyi et al., 2016). However, excessive consumption of isoleucine can cause side effects such as diarrhea and mental disorders. A diet containing excessive leucine can also increase the amount of ammonia in the body and damage the liver and kidney functions. Our study shows that isoleucine is a risk factor for depression, and the risk of depression increases with isoleucine exposure. Therefore, in the daily diet, attention should be paid to isoleucine intake.

Glutamine is not an essential amino acid, but it is considered a conditionally essential amino acid, particularly under catabolic stress. Glutamine metabolism affects the process of depression (Liu et al., 2021). As an amino acid analog, glutamine can protect and repair gastrointestinal mucosa as well as improve the brain function of children with intellectual disabilities and patients with alcoholism, mental disorders, and epilepsy. Our study indicated that glutamine had also a protective effect on depression (OR 0.9845, 95% CI 0.9718–0.9974; *P*-value = 0.0188), which was consistent with previous studies (Özkan et al., 2021). Glutamine is catalyzed by glutaminase in neurons to synthesize glutamate, which is then released into synaptic gaps to exert biological effects. Under the action of glutamate transporters, the released glutamate is taken up by the surrounding astrocytes, re-metabolized to glutamine, and subsequently transported to the presynaptic neurons for conversion to glutamate. In addition, glutamic acid can be metabolized to gamma-aminobutyric acid by glutamic acid decarboxylase. Therefore, the glutamine–glutamine–gamma–aminobutyric acid cycle plays a role in glial cell communication during excitatory and inhibitory neurotransmission. Many studies have shown that abnormal glutamine–glutamine circulation plays an important function in the development of depression (Chen et al., 2019). The glutamate–glutamine cycle is formed by the mutual conversion of glutamate and glutamine under physiological conditions, which involves a variety of receptors and transporters, and it is a potential target for the development of novel antidepressant drugs (Chen et al., 2019). Furthermore, glutamine has a good effect on balancing the intestinal microbiomes. It increases the expression of tight junction protein and the integrity of the intestinal lining as well as minimizes the inflammatory response in the presence of intestinal mucosal irritation. The intestinal function is regulated by the tenth cranial nerve or the vagus nerve, which directly affects neurotransmitter balance. Depression is associated with suboptimal microbiomes, unhealthy intestinal permeability, and inflammation. an inflamed gut has a direct influence on neurotransmitter balance and brain health. Glutamine reduces depression by maintaining intestinal health, protecting the enteric nervous system, protecting the intestinal lining from damage, and suppressing chronic inflammation (Deters and Saleem, 2021).

The most direct energy source of human life activity Is glucose. Autopsy studies have found that the mitochondrial separation of hexokinase 1 in the cerebral parietal cortex tissue of patients with depression is significantly increased, leading to brain tissue cell swelling and toxicity (Regenold et al., 2012). Therefore, abnormal glucose metabolism is observed in patients with depression. Further research shows that suicide attempts in men and women were significantly related to the body's blood sugar levels (Dong et al., 2021), which is consistent with the results of this research. At present, the association between depression and glucose metabolism disorder has been widely studied and recognized, but whether the two diseases co-occur or which disease is the cause of the other remains unclear (Réus et al., 2017). Our study found that glucose is positively associated with depression, a risk factor for depression, and reverse MR analysis showed no significant correlation between depression and glucose levels.

As the main apolipoprotein of HDL cholesterol, apolipoprotein A-I has an important function in the reversal of cholesterol transport and phospholipid and lipoprotein metabolism (Wang and Rader, 2007). Given its small molecular size, apolipoprotein A-I can cross the blood–brain barrier, and it is considered a major component of HDL (Lee et al., 2021). This study found that apolipoprotein A-I is negatively correlated with depression, which is a protective factor for depression, and it could be a promising target for depression treatment Therefore, future studies can strengthen the research on the role of apolipoprotein A-I in depression.

As an end product of protein metabolism, the toxicity of urea has long been considered negligible. A meta-analysis of the association between depression and chronic kidney disease (CKD) has shown that depression is due to damage to the medial prefrontal cortex as found in the mice model of CKD and a cohort of CKD patients. In the mammalian kidneys, urea plays an important role in water conservation and urine concentration, and it is also the end product of protein metabolism. Bypassing psychosocial stress, the buildup of urea in the brain serves as an independent factor that causes depression (Wang et al., 2019). Expanded cohort studies have shown that urea is associated with depression. Urea can cause depression, interrupt long-term potentiation, and induce synaptic loss in mice models. Inhibition of the mTORC1-S6K pathway is necessary for the action of urea, and cyanate, as a hydrolyzed product of urea, is also involved in this pathophysiological process. The results suggest that the accumulation of urea in the brain is an independent contributor to depression. Carbamylated mTOR with urea or cyanate inhibits Mtorc1-S6K-dependent dendrite protein synthesis, causing damage to mPFC synaptic plasticity and depressive behavior (Wang et al., 2019). Urea accumulation in the hippocampus induced by the loss of the urea transporter also causes depression-like behavior (Li et al., 2012). Furthermore, the increase of urea concentration in the body can activate NF-κ and AMPK-related signaling pathways and then induce inflammation in human microvascular endothelial cells. The expression of NOS in endothelial cells is also affected by the stimulation of high-urea concentration, which leads to the change of NO content in cells, thereby affecting the function of endothelial cells. This study found that urea is positively correlated with depression. Thus, it is considered a risk factor for depression. Therefore, urea accumulation in the brain should be prevented to prevent and treat depression, promote the timely discharge of urea, and avoid the occurrence of urea metabolism disorders. In addition, the glycoprotein is a risk factor for depression, but few studies have been conducted on glycoprotein in depression. Future studies on the function and mechanism of glycoprotein in depression should be strengthened, which may be a potential target for anti-depression.

The cohorts in genome-wide studies of circulating metabolites included 37–64% of women in the sample demographics, and genome-wide studies of depression phenotypes included 53–56% of women in the sample population, which indicated that women are evenly represented. Thus, our findings are also applicable to women with depression. This study also has several limitations. First, important covariates such as diet, drug use, and environmental impact could not be adjusted although the use of MR-excluded confounding factors increased the accuracy and reliability of the study as well as yielded interesting results. Second, the two GWASs adopted the meta-analytic method by pooling data from several cohorts. This approach performs well in obtaining genetic characteristics of the general population, but it may lead to the deviation of sample overlap. Third, MR analyses typically reveal lifetime exposure, thus a further study should be carried out in randomized controlled trials on the effects of exposure. Finally, participants involved in the two GWASs were primarily of European descent; thus, our results may be applicable to populations of European descent. It should be cautious to generalize the findings to other ethnic groups, and further research is needed.

Collectively, our MR study suggests a protective causal effect of glutamine and apolipoprotein A-I on depression. In addition, our study found that acetoacetate, isoleucine, glucose, glycoproteins, and urea are risk factors for depression. Reverse MR analysis showed that depression had no effect on these seven metabolites. Compared with previous studies, this study found no significant causal relationship between alanine, histidine, tyrosine, creatinine, etc., and depression, and these findings are worthy of an in-depth study.

## Data availability statement

The original contributions presented in the study are included in the article/Supplementary material, further inquiries can be directed to the corresponding authors.

## Author contributions

YD designed the study and wrote the manuscript. ZZ collected the data. PD analyzed the data. XF and CL reviewed the manuscript. All authors approved the final manuscript.
