# Supplementary material for: Circulating metabolites and depression: a bidirectional Mendelian randomization
Source: Front Neurosci. 2023 Apr 21;17:1146613. doi: 10.3389/fnins.2023.1146613 (PMC10160621; doi:10.3389/fnins.2023.1146613)

Supplementary Figure 1. Forest plots of each metabolite associated with depression. A. Acetoacetate. B. Apolipoprotein A-I. C. Glucose. D. Glutamine. E. Glycoproteins. F. Isoleucine. G. Urea.


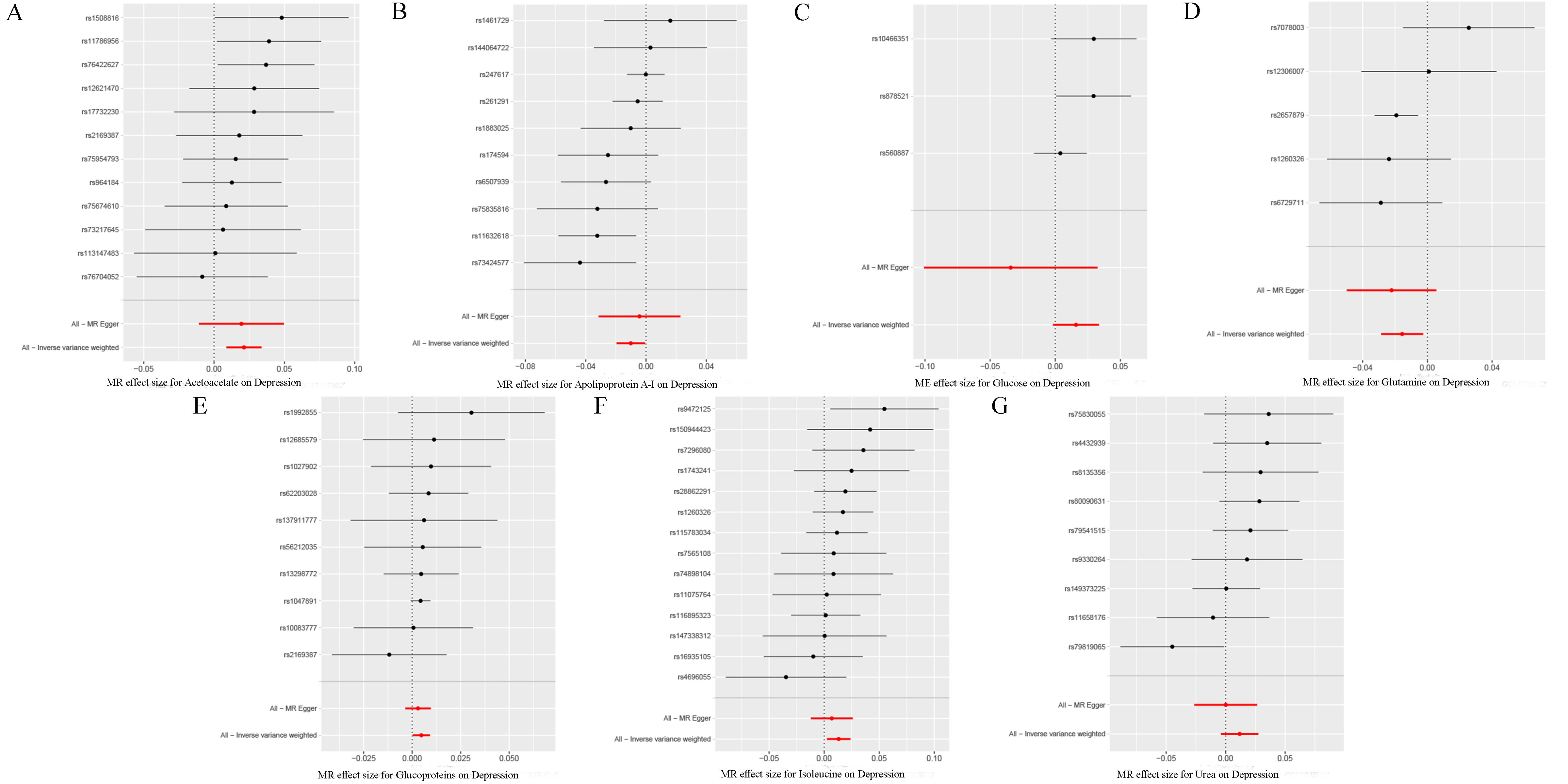


Supplementary Figure 2. Funnel plots to visualize overall heterogeneity of MR estimates for the effect of each metabolite on depression. A. Acetoacetate. B. Apolipoprotein A-I. C. Glucose. D. Glutamine. E. Glycoproteins. F. Isoleucine. G. Urea.


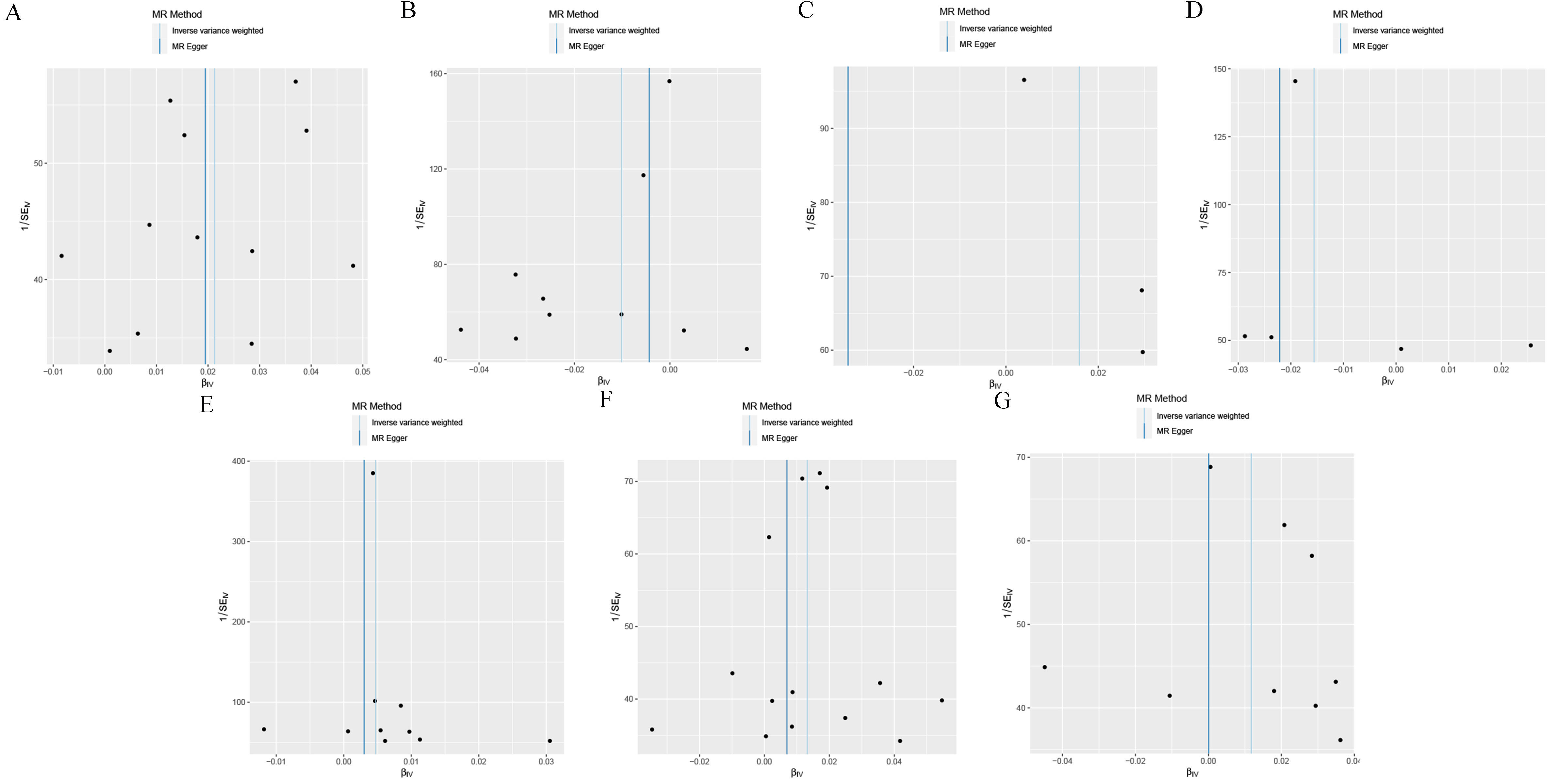

Supplement: Supplementary file 1 [file Data_Sheet_1.docx]
